# Virulent Clones of *Klebsiella pneumoniae*: Identification and Evolutionary Scenario Based on Genomic and Phenotypic Characterization

**DOI:** 10.1371/journal.pone.0004982

**Published:** 2009-03-25

**Authors:** Sylvain Brisse, Cindy Fevre, Virginie Passet, Sylvie Issenhuth-Jeanjean, Régis Tournebize, Laure Diancourt, Patrick Grimont

**Affiliations:** 1 Institut Pasteur, Genotyping of Pathogens and Public Health, Paris, France; 2 Institut Pasteur, Biodiversité des Bactéries Pathogènes Emergentes, Paris, France; 3 Institut Pasteur, Unité de Pathogénie Microbienne Moléculaire, Paris, France; 4 Unité INSERM U786, Institut Pasteur, Paris, France; Institut de Pharmacologie et de Biologie Structurale, France

## Abstract

*Klebsiella pneumoniae* is found in the environment and as a harmless commensal, but is also a frequent nosocomial pathogen (causing urinary, respiratory and blood infections) and the agent of specific human infections including Friedländer's pneumonia, rhinoscleroma and the emerging disease pyogenic liver abscess (PLA). The identification and precise definition of virulent clones, i.e. groups of strains with a single ancestor that are associated with particular infections, is critical to understand the evolution of pathogenicity from commensalism and for a better control of infections. We analyzed 235 *K. pneumoniae* isolates of diverse environmental and clinical origins by multilocus sequence typing, virulence gene content, biochemical and capsular profiling and virulence to mice. Phylogenetic analysis of housekeeping genes clearly defined clones that differ sharply by their clinical source and biological features. First, two clones comprising isolates of capsular type K1, clone CC23^K1^ and clone CC82^K1^, were strongly associated with PLA and respiratory infection, respectively. Second, only one of the two major disclosed K2 clones was highly virulent to mice. Third, strains associated with the human infections ozena and rhinoscleroma each corresponded to one monomorphic clone. Therefore, *K. pneumoniae* subsp. *ozaenae* and *K. pneumoniae* subsp. *rhinoscleromatis* should be regarded as virulent clones derived from *K. pneumoniae*. The lack of strict association of virulent capsular types with clones was explained by horizontal transfer of the *cps* operon, responsible for the synthesis of the capsular polysaccharide. Finally, the reduction of metabolic versatility observed in clones Rhinoscleromatis, Ozaenae and CC82^K1^ indicates an evolutionary process of specialization to a pathogenic lifestyle. In contrast, clone CC23^K1^ remains metabolically versatile, suggesting recent acquisition of invasive potential. In conclusion, our results reveal the existence of important virulent clones associated with specific infections and provide an evolutionary framework for research into the links between clones, virulence and other genomic features in *K. pneumoniae*.

## Introduction

*Klebsiella pneumoniae* is responsible for a variety of diseases in humans and animals [1]–[3]. Most notoriously, *K. pneumoniae* is a prominent nosocomial pathogen mainly responsible for urinary tract, respiratory tract or blood infections [4]. Isolates from hospitals often display antibiotic resistance phenotypes [5], [6], while resistance isolates and genetic elements may also spread into the community [7], [8]. Nosocomial infections are caused by highly diverse *K. pneumoniae* strains that may be considered as opportunistic, rather than true pathogens, since they mostly affect debilitated patients [4]. In contrast, serious community infections due to *K. pneumoniae* can affect previously healthy persons. Historically, *K. pneumoniae* was described as the agent of Friedländer's pneumonia, a severe form of lobar pneumonia with a high mortality [9]. *K. pneumoniae* is still one of the leading causes of community acquired pneumoniae in some countries [10], [11]. Recently, *K. pneumoniae* pyogenic liver abscess (PLA), sometimes complicated by endophthalmitis or meningitis, emerged in Taiwan and other Asian countries, as well as in other continents [12]–[17]. Rhinoscleroma and atrophic rhinitis (also called ozaena) are two chronic and potentially severely disturbing diseases of the upper respiratory tract, associated respectively with *K. pneumoniae* subsp. *rhinoscleromatis* and *K. pneumoniae* subsp. *ozaenae* [3], [18]–[21]. Other *K. pneumoniae* infections that are severe but more rarely reported include meningitis, necrotizing fasciitis and prostatic abscess [22]–[24]. Finally, granuloma inguinale (donovanosis) [25] is caused by uncultivated bacteria, which may belong to *K. pneumoniae* [26], [27].

Factors that are implicated in the virulence of *K. pneumoniae* strains include the capsular serotype, lipopolysaccharide, iron-scavenging systems, and fimbrial and non-fimbrial adhesins [3], [28]–[31]. The abundant polysaccharidic capsule that typically surrounds *K. pneumoniae* protects against the bactericidal action of serum and impairs phagocytosis, and may be regarded as the most important virulence determinant of *K. pneumoniae*. Among the 77 described capsular (K) types of the serotyping scheme, types K1, K2, K4 and K5 are highly virulent in experimental infection in mice and are often associated with severe infections in humans and animals [1], [32]–[35]. K1 isolates were frequent among Friedländer's peumonia cases [1], [36] and are prominent among PLA cases, especially those with complications. Serotypes K2, K4 and K5 are frequent causes of metritis in mares and were also associated with community-acquired pneumonia [1], [36]. Isolates causing rhinoscleroma are always of type K3 [18], [27]. Finally, although their role as a direct cause of ozaena is not fully established, *K. pneumoniae* subsp. *ozaenae* isolates from cases of atrophic rhinitis are of serotype K4 or more rarely K5 [18].

In contrast with the extensive knowledge that has been gathered on the genotype-virulence relationships in the closely related species *Escherichia coli and Salmonella enterica*, virulent clones of *K. pneumoniae* remain virtually undefined [31], [37], [38]. Critically, it is unknown whether particular diseases are caused by specific clones or rather, by the expression of particular virulence determinants. This distinction is important, as virulence factors may be horizontally transferred among strains and could be weakly associated with the genomic background that harbor them, with clear implications for emergence of new pathogens and for diagnostic purposes. It is currently unknown whether capsular types characterize specific clones, in which case the K type may be useful to identify such clones and to predict the presence of other associated virulence determinants. Alternately, as is the case in e.g. *Streptococcus pneumoniae* [39], K types may be distributed across many unrelated clones due to frequent horizontal transfer of the *cps* operon, which is responsible for the synthesis of the capsular polysaccharide. In this case, a more complex picture is to be expected for the association of capsular types, other virulence determinants, and strain genomic background. More generally, the genetic structure of *K. pneumoniae* remains virtually unexplored [40], [41], and the phylogenetic relationships among virulent strains causing identical or distinct diseases are therefore unknown. In addition, the relationships between environmental, carriage or virulent *K. pneumoniae* isolates are undocumented. As a consequence, limited information on how these strains evolved to become pathogenic is currently available.

Evolution towards increased virulence can be accompanied by ecological changes that reflect specialization of pathogenic bacterial clones to their new lifestyle. For example, evolution of the particular pathogenic pattern of *Shigella* or *Salmonella enterica* serotype Typhi has been paralleled by host restriction and reduction of metabolic capabilities [42]–[44]. With the exception of the well-known reduced metabolic capabilities of *K. p.* subsp. *rhinoscleromatis* and *K. p.* subsp. *ozaenae* [27], it is not known whether the virulent strains of *K. pneumoniae* belong to ecologically specialized pathogenic clones.

The purposes of this study were (i) To determine the population genetic structure of *K. pneumoniae*, with a particular emphasis on the definition of virulent clones and their distinctness from other strains; (ii) To determine the extent of horizontal transfer of capsular synthesis (*cps*) operons among clones; and (iii) To characterize the virulent clones with respect to capsular type, other known virulence factors, experimental virulence to mice, and metabolic properties.

## Results

### 1. Restricted levels of genetic diversity and recombining population structure

Alignment of the seven genes sequences from 235 isolates showed no insertion/deletion (indel) in six genes. In gene *tonB*, one insertion of two codons (isolate SB3336) and three deletion events (one of four codons, and two of two codons) were observed. Excluding these four indels, 129 (4.3%) of the 3,012 nucleotides positions were polymorphic, four of them corresponding to tri-allelic single nucleotide polymorphisms (SNPs), thus implying a total of 133 mutations. The maximal level of nucleotide divergence among alleles ranged from 0.37% (*gapA*) to 1.74% (*phoE*), while the diversity index π (the average number of nucleotide differences per site between any two sequences chosen randomly from the study sample) ranged from 0.14% (for *gapA*) to 1.0% (for *tonB*) (**Table 1**). Synonymous substitutions were 12 times more frequent than non-synonymous substitutions. Despite this high degree of sequence conservation, a total of 117 haplotypes or sequence types (STs) were distinguished.

**Table 1:** Nucleotide polymorphism among 235 *Klebsiella pneumoniae* isolates.

Gene	Size	No. (%) of polymorphic sites	No. of synonymous sites	No. of non-synonymous sites	Ks	Ka	Ka/Ks	π
*gapA*	450	13 (2.9)	13	0	0.00563	0.000	0.000	0.00142
*infB*	318	17 (5.3)	15	2	0.01381	0.00007	0.0051	0.00309
*mdh*	477	21 (4.4)	16	6	0.00697	0.00055	0.079	0.00219
*pgi*	432	20 (4.6)	19	1	0.0052	0.00043	0.083	0.00157
*phoE*	420	25 (6.0)	20	5	0.02842	0.00055	0.019	0.00705
*rpoB*	501	14 (2.8)	11	3	0.00288	0.00136	0.47	0.00174
*tonB*	414	21 (5.1)	13	8	0.02739	0.00415	0.15	0.01005
concatenate	3,012	129 (4.29)	103	26	0.01192	0.00099	0.083	0.0037
*gnd* (a)	360	136 (37.8)	142	11	0.208	0.0043	0.021	0.055

(a) Only 177 strains were sequenced.

Ks: No. of synonymous changes per synonymous site. Ka: No. of non-synonymous changes per non-synonymous site.

π: nucleotide diversity.

**Figure 1:**
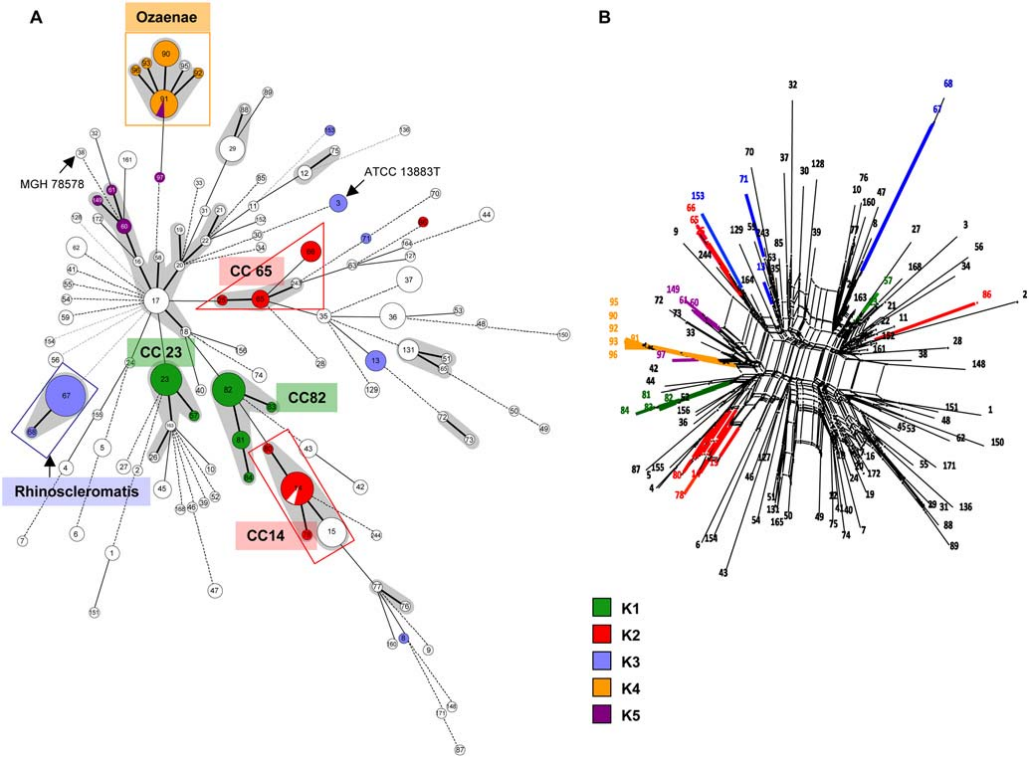
Clonal diversity and relationships among 235 *Klebsiella pneumoniae* isolates. A. Minimum spanning tree (MStree) analysis of multilocus sequence typing (MLST) data for 235 *K. pneumoniae* isolates, representing 117 sequence types (STs). Isolates of capsular serotypes K1 to K5 are colored according to serotype. Each circle corresponds to a sequence type (ST); ST number is given inside each circle. Grey zones surround STs that belong to the same clonal complex (CC), which is named according to the central ST (the likely founder of the CC). CC65-K2 is delimited by the red triangle (see text). The lines between STs indicate inferred phylogenetic relationships and are represented as bold, plain, discontinuous and light discontinuous depending on the number of allelic mismatches between profiles (1, 2, 3 and 4 or more, respectively); note that discontinuous links are only indicative, as several alternative links with equal weight may exist. The STs of reference genome strain MGH78578 (ST38) and of the type strain ATCC 13883T (ST3) are indicated. B. Split decomposition analysis of concatenated sequences of the seven genes. Numbers at the tip of branches are ST numbers. Note the bushy network structure indicative of pervasive homologous recombination. Branches were colored for the clones that are highlighted on panel A. Note the distribution into unrelated branches of strains with a given capsular (K) type.

Visual inspection of the repartition of polymorphic sites across the phylogeny of the concatenated sequence suggested that many polymorphisms have been shuffled by genetic exchange. The strong network structure obtained after split decomposition analysis (**Figure 1B**) confirmed the high level of incompatibility among sites, indicative of a pervasive history of intra- and/or intergenic recombination. Recombination was detected by LDhat with statistical significance in the two most polymorphic genes, *tonB* (r/m ratio, 22.3; p = 0.02) and *phoE* (r/m ratio, 18.1; p = 0.0084), indicative of intragenic recombination in these genes. Frequent polymorphisms were too scarce (no more than 1 or 2 polymorphisms above the 0.1 frequency level) in the remaining five genes to test for recombination. Evidence of homologous recombination was also provided by the observation of multiple nucleotide substitutions between STs differing by a single allele (single locus variants, or SLVs). First, ST16 and ST60 differed only by six nucleotides at gene *tonB* (alleles *tonB*-4 and *tonB*-8). Second, ST65 and ST243 were identical except for five SNPs between their alleles *tonB*-13 and *tonB*-25. For these two cases, import of a recombining segment, rather than independent mutations in the mismatched gene, seems compelling. In total, eight allelic mismatches (24%) between SLV pairs involved more than one SNP and are likely to result from genetic exchange. Of note, single nucleotide changes may also have been introduced by homologous recombination, given the very high sequence relatedness among most alleles. In conclusion, recombination appears frequent among housekeeping genes in *K. pneumoniae*.

### 2. Identification of virulent clones of *K. pneumoniae*

As expected given frequent recombination and low levels of sequence divergence, sequence-based phylogenetic analysis using PhyML [45] revealed a bushy tree (data not shown) with no conspicuous internal structure and only few strongly supported nodes. ClonalFrame [46] failed to estimate the population parameters, probably due to insufficient polymorphism. The splits decomposition network (**Figure 1B**) revealed no internal phylogenetic structure, with few obvious haplotype associations. In order to reveal relationships among closely related haplotypes with an approach that is less sensitive to recombination, we used the minimum spanning tree (MStree) method based upon allelic profiles. When allowing only one allelic mismatch to assign isolates to a given clonal complex (CC), 12 CCs were disclosed (the same groups were identified using eBURST [47]), while the remaining isolates were distributed into 72 singletons (**Figure 1A**).

Remarkably, six CCs corresponded to serotypes K1 to K4, and these CCs were characterized by their distinctive K type or pathological origins. Notably, K1 isolates were distributed into two CCs, CC23^K1^ and CC82^K1^ (**Figure 1A**). CC23^K1^ was composed of ST23 and ST57, which comprised only K1 isolates, and ST26 and ST163, which included K35 and K61 isolates. Differently, CC82^K1^ included only K1 isolates. Remarkably, the four K1 isolates from cases of PLA belonged to CC23^K1^: three isolates from Taiwan belonged to ST23 and one isolate from Zaire had ST57. Other isolates of ST23 were isolated from horses and from human blood infections (four isolates each; see **Table S1**). CC82^K1^ comprised the 15 remaining K1 isolates, none of which was involved in liver abscess. These isolates included the reference strain of serotype K1 (A5054, ST82) as well as 11 isolates from blood or respiratory infection in France between 1976 and 1984.

Likewise, all K2 isolates except CIP 52.204 (ST86) could be grouped into two distinct and apparently unrelated groups (**Figure 1A**). First, CC14^K2^ comprised STs 14, 78 and 80, all being composed of isolates of serotype K2, and also included some K24 isolates (ST15). ST14 included ESBL-producing isolates from Curaçao [48] and isolates collected in France and Italy between 1981 and 2002 from urinary, respiratory, blood and cerebro-spinal fluid (CSF) infections. A second group of K2 isolates was composed of STs 65, 25 and 243, which together formed one CC, and of ST66, which differed from ST65 by only two genes (*infB* and *rpoB*, one SNP each). ST65 includes an isolate from a cat infection, one isolate that caused an epidemics in monkeys at a French zoo [49], and one human clinical isolate from an anal abscess, while ST25 corresponds to a nosocomial blood isolate (**Table S1**). ST66 corresponds to the reference strain of capsular serotype K2 (B5055) and the virulent strain CIP 52.145 [34], as well as to isolate 675, which was used as a vaccine in animals (**Table S1**). For simplicity, and given the genetic and phenotypic similarity of ST66 strains with ST65 strains, we will consider ST66 as a member of CC65^K2^, even if ST66 does not belong to CC65^K2^ *sensu stricto*.

Sets of isolates which belong to a single clonal complex and share many other common features including K type, virulence factor content and metabolic profile (see below), likely descend from a common ancestral strain from which they inherited their common properties. Therefore, CC23^K1^, CC82^K1^, CC14^K2^ and CC65^K2^ may be regarded as four distinct clones. However, their precise demarcation is rendered difficult, based on MLST data alone, by the high degree of allele sharing with other *K. pneumoniae* STs, possibly due to their recent evolutionary emergence or to ongoing allelic exchange with other STs (see discussion).

All *K. pneumoniae* subsp. *rhinoscleromatis* isolates were identical at the seven genes (ST67) except one isolate (839, France, 1982), which had a single SNP in *tonB*, resulting in ST68 (**Figure 1A**). ST67 included CIP 52.210^T^, the type strain of *K. pneumoniae* subsp. *rhinoscleromatis*, and C5046, the reference strain of capsular serotype K3. In addition, ST67 included nine unrelated clinical isolates from rhinoscleroma cases, isolated from six countries between 1954 and 2003. Clearly, subspecies *K. pneumoniae* subsp. *rhinoscleromatis* is highly homogeneous and appears to correspond to a single clone, which we refer to as clone Rhinoscleromatis. Interestingly, all Rhinoscleromatis isolates differed from all other strains, including 10 non-rhinoscleroma isolates with serotype K3, by four or more allelic mismatches. MLST thus clearly demarcates Rhinoscleromatis isolates from all other *K. pneumoniae* members. However, it is important to stress that Rhinoscleromatis clearly belongs to a single genetic pool together with *K. pneumoniae* (**Figure 1B**): the average genetic distance between Rhinoscleromatis and the 115 other STs is 0.54%, while distances among the 115 STs ranged from 0.033% to 0.70%.

All *K. pneumoniae* subsp. *ozaenae* isolates formed a single clonal complex, CC91^oz^. ST91 could be inferred as the genetic founder of this clone (**Figure 1A**), as all other STs of CC91^oz^ differed from ST91 by a single mismatch, whereas they differed among them by two mismatches, with the single exception of the pair ST95 and ST96. ST91 included CIP 52.211^T^, the type strain of *K. pneumoniae* subsp. *ozaenae*, and the K4 reference strain D5050, as well as the reference strain of type K5 (CIP 52.212 = E5051) and two clinical isolates. The other STs of CC91^oz^ included K4 clinical isolates from ozena cases and blood infections, as well as two isolates from patients with granulomas (ST90 and ST96). Isolates from ozaena cases were distributed in the three genotypes ST90, ST91 and ST95. These results indicate that all *K. pneumoniae* subsp. *ozaenae* isolates can be considered as descending from a single ancestor, forming clone Ozaenae. This clone was well demarcated from the remaining isolates, as there was only one *K. pneumoniae* strain (SB169-2, ST97, C-pattern C16a) that had only two allelic mismatches with clone Ozaenae, while all other *K. pneumoniae* isolates, including three non-Ozaenae K5 isolates, had at least four mismatches with any member of clone Ozaenae. Of note, clone Ozaenae (6 STs) is more heterogeneous than clone Rhinoscleromatis (2 STs) based on the present strain collection, possibly reflecting a more ancient evolutionary emergence and/or a more rapid diversification.

### 3. Capsular types are not strongly associated with genomic background

**Figure 2:**
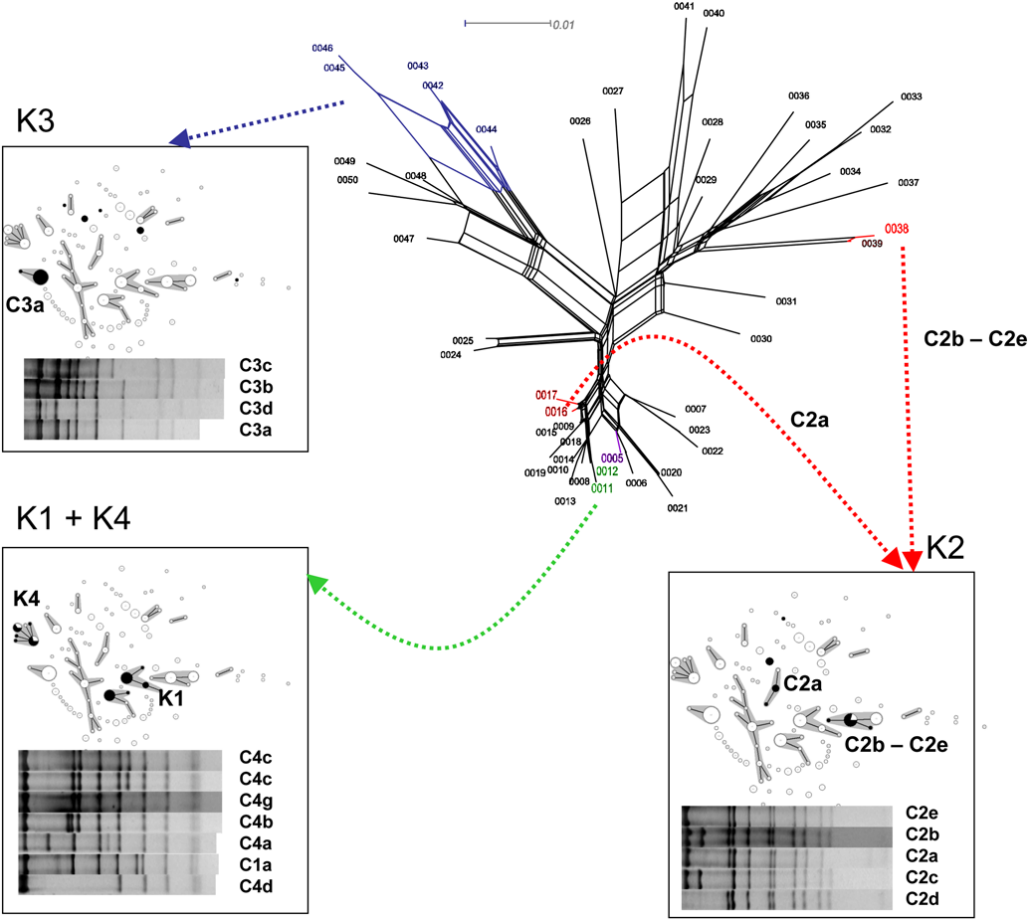
Distribution of related capsular operon regions in unrelated clones. The splits tree represents the relationships among *gnd* alleles as obtained after split decomposition analysis. The distribution of the *gnd* alleles found in isolates and reference strains of capsular serotypes K1 to K4 is indicated by black coloration of sequence types (STs) in the MStree of the corresponding insets. Below the MStree displays are represented the C-pattern of the corresponding isolates. Note that similar or identical *gnd* and C-patterns are distributed in unrelated STs.

No close phylogenetic relatedness was apparent between the two K1 groups, between the two K2 groups, and between clones Rhinoscleromatis and Ozaenae with other *K. pneumoniae* strains of serotypes K3, K4 or K5 (**Figure 1**), indicating an independent origin in distinct genomic backgrounds, rather than a common ancestral origin. Two evolutionary mechanisms could result in identical K-types being distributed in unrelated genomic backgrounds: horizontal transfer of the *cps* operon, or evolutionary convergence. In the latter scenario, similar capsular polysaccharide antigenic structures would be synthesized by phylogenetically unrelated *cps* operons that are functionally identical. In order to estimate the phylogenetic relatedness of *cps* operon structures, isolates were analyzed by PCR-RFLP of the *cps* operon [50], which can disclose unrelated C-patterns among isolates of a given K type. The C-pattern of 211 isolates could be established (**Table S1**; C-patterns available upon request). Clearly, indistinguishable or highly similar C-patterns were observed in unrelated MLST genotypes (**Figure 2**): K1 isolates from both CC23^K1^ and CC82^K1^ had C-pattern C1a, while K2 isolates of CC14^K2^ and CC65^K2^ exhibited the highly similar patterns C2b to C2e (CC14^K2^) and C2a (CC65^K2^). All K3 isolates from clone Rhinoscleromatis and from the 10 K3 *K. pneumoniae* isolates in five other STs, had C-pattern C3a or the highly similar patterns C3b to C3d (**Figure 2**). In particular, C3a was observed in all Rhinoscleromatis isolates as well as in the unrelated ST3 and ST13. The three variant K3 C-patterns were observed in ST8 (C3c), ST71 (C3b) and ST153 (C3d). Likewise, the C-pattern C5a was observed in clone Ozaenae K5 isolates and in *K. pneumoniae* K5 isolates (ST60, ST61 and ST149), which do not appear phylogenetically related (**Figure 1**). Altogether, these data are suggestive of several independent historical events of horizontal transfer of the *cps* operon between isolates belonging to distinct clones.

In order to fully demonstrate suspected cases of *cps* region horizontal transfer, we sequenced in 177 relevant isolates, a 360-nt internal portion of gene *gnd*, which genomic location is just adjacent of the *cps* operon [50], [51]. A high level of nucleotide polymorphism was encountered (**Table 1**), with 136 (38%) polymorphic sites, with no indel. Thirteen isolates had a *gnd* sequence that differed from the 164 other sequences by 6% to 18%. The remaining 164 sequences were still much more variable than the seven MLST genes. LDhat analysis demonstrated a strong intra-genic recombination pattern (r/m ratio = 12.5, p = 0.008), consistent with the highly reticulated structure obtained using SplitsTree (**Figure 2**). However, despite frequent intra-genic recombination, *gnd* alleles were remarkably similar for isolates of the same K type (**Figure 2**), indicating that the association between the *gnd* gene and the *cps* operon was not broken down.

Notably, the *gnd* sequences of K1 isolates from CC23^K1^ and CC82^K1^ were undistinguishable (*gnd*-12 in both CCs, and *gnd*-11 in two strains of ST82, differing at a single SNP from *gnd*-12). Similarly, the *gnd* sequence of Rhinoscleromatis isolates (alleles *gnd*-42, *gnd*-45 and *gnd*-46) were either identical or highly similar (**Figure 2**) to those of *K. pneumoniae* K3 isolates (*gnd*-42, *gnd*-43 and *gnd*-44), demonstrating their recent common ancestry and their horizontal transfer into distinct genomic backgrounds. Likewise, K5 isolates of clone Ozaenae and K5 *K. pneumoniae* isolates (ST60 and ST61) had identical *gnd* sequences (*gnd*-5). Together with identity of C-patterns, these data demonstrate a common evolutionary origin of the *cps-gnd* region in Rhinoscleromatis and K3 *K. pneumoniae* isolates, in Ozaenae and K5 *K. pneumoniae* isolates, and in both K1 groups. Horizontal gene transfer of entire *gnd-cps* region is the most likely explanation for the current distribution of *cps-gnd* regions with a unique origin in distinct genomic backgrounds. The transfer of the *gnd-cps* region could be inferred for other K types as well (data not shown).

Different from the above, *gnd* sequences in K2 isolates of CC14^K2^ (*gnd*-38) and CC65^K2^ (*gnd*-16 or *gnd*-17) were unrelated (**Figure 2**). Because the C-pattern from these two CCs are highly related (**Figure 2**), it is likely that the *cps* operon was transferred horizontally without the *gnd* gene, or that the *gnd* gene was replaced in one of the CCs subsequently to a *cps-gnd* co-transfer event. However, as allele *gnd*-38 was also observed in K2 strain CIP 52.204 (ST86), horizontal transfer of the *cps-gnd* region has occurred between this ST and CC14^K2^.

It is remarkable that the *gnd* allele in K4 isolates of clone Ozaenae was the same as observed in most K1 isolates (*gnd*-12). This is fully consistent with the observation that K1 and K4 C-patterns (**Figure 2**) are highly similar [50] and indicates a close evolutionary relationships of the *cps* operons that determine K1 and K4 serotypes.

### 4. Virulence is associated with clone, rather than with K-type

In order to determine whether the above-identified clones differ by their virulence potential, the presence of 10 genetic factors implicated in *Klebsiella* virulence was assessed by PCR. A total of 102 representative isolates were characterized (**Table 2**). While the three genes *uge*, *wabG* and *ureA* gave a positive PCR reaction in all isolates, other factors showed unequal repartition across CCs, resulting in distinctive virulence factor fingerprints of major CCs (**Table 2**). Notably, we found sharp differences in virulence gene content between CC23^K1^ and CC82^K1^, as well as between CC14^K2^ and CC65^K2^. Consistent with the location of *magA* within the *cps* operon of K1 isolates [52], both K1 groups were *magA* positive, while *magA* was not detected in any other isolate. However, CC23^K1^ differed from CC82^K1^ by the presence (100% vs. 0%, respectively) of genes *mrkD* coding for the type 3 fimbriae adhesin, which facilitates adhesion to the basement membranes of several human tissues [53], [54], and *allS*, coding for the activator of the allantoin regulon [30]. Interestingly, *allS* was specific for K1 isolates of CC23^K1^ members, as it was undetected in CC82^K1^ and in non-K1 members of CC23^K1^ (ST26-K61 and ST163-K35). CC23^K1^ was also characterized by a higher prevalence (80%) of non-fimbrial adhesin CF29K [55], whereas CC82^K1^ and most other isolates were negative.

**Table 2:** Virulence gene content of *Klebsiella pneumoniae* clones (a).

Gene	clone Rhinoscleromatis (n = 13)	clone Ozaenae (n = 12)	CC23K1 (n = 10) (b)	CC82K1 (n = 15)	CC14K2/K24 (n = 20)	CC65K2 (n = 9)
*magA*	0	0	100	100	0	0
*allS*	0	0	100	0	0	0
*rmpA*	100	41.7	80	86.7	0	77.8
*mrkD*	100	8.3	100	0	100	88.9
*kfu*	0	50	100	100	100	0
*cf29a*	0	8.3	80	0	0	44.4
*fimH*	100	100	100	93.3	100	88.9
*uge*	100	100	100	100	100	100
*wabG*	100	100	100	100	100	100
*ureA*	100	100	100	100	100	100

(a) The number of tested strains is given in parentheses after ‘n = ’. Values are % of strains with positive PCR reaction.

(b) Only K1 strains of CC23K1 are considered.

The two K2 groups CC14^K2^ and CC65^K2^ also differed by their virulence gene content. Particularly, isolates of CC14^K2^, including its K24 members, were all positive for the iron uptake marker *kfu* [31], whereas all CC65^K2^ isolates were negative. In contrast, *rmpA*, the regulator of mucoid phenotype [56], was undetected in CC14 whereas *rmpA* PCR was positive in 71% of CC65^K2^ isolates (**Table S1**).

Clone Rhinoscleromatis was characterized by the complete absence of *kfu* and the presence of *rmpA*. These characteristics also distinguished Rhinoscleromatis from other K3 *K. pneumoniae* isolates (**Table S1**). Ozaenae isolates shared the unique property, together with CC82^K1^, of being negative for *mrkD* (except for one isolate).

To determine whether the two K1 clones and the two K2 clones differ in their virulence, four to nine strains per clone were tested in mice (**Table S1**). There was a clear difference in the virulence of CC14^K2^ and CC65^K2^, as no strain (0 out of nine) of the former was lethal, whereas four out of six CC65 strains killed mice after five days. The two avirulent CC65 strains were either *rmpA* negative (as were all CC14 K2 strains) or negative for *fim* and *mrkD* (**Table S1**). Likewise, out of seven CC23^K1^ strains, four K1 strains (ST57 and three of ST23; all *rmpA* positive) were lethal to mice. The three avirulent strains were one ST23 K1 strain and the two non-K1 strains of ST26 and ST163; these three strains lacked *rmpA*. In contrast, of the four CC82^K1^ strains assayed, only one was slightly virulent to mice, even though *rmpA* PCR was positive (**Table S1**). Hence, virulence to mice of K1 and K2 strains appeared to differ, depending on the clone they belonged to.

### 5. Metabolic versatility and evolution of virulent *K. pneumoniae* clones

**Figure 3:**
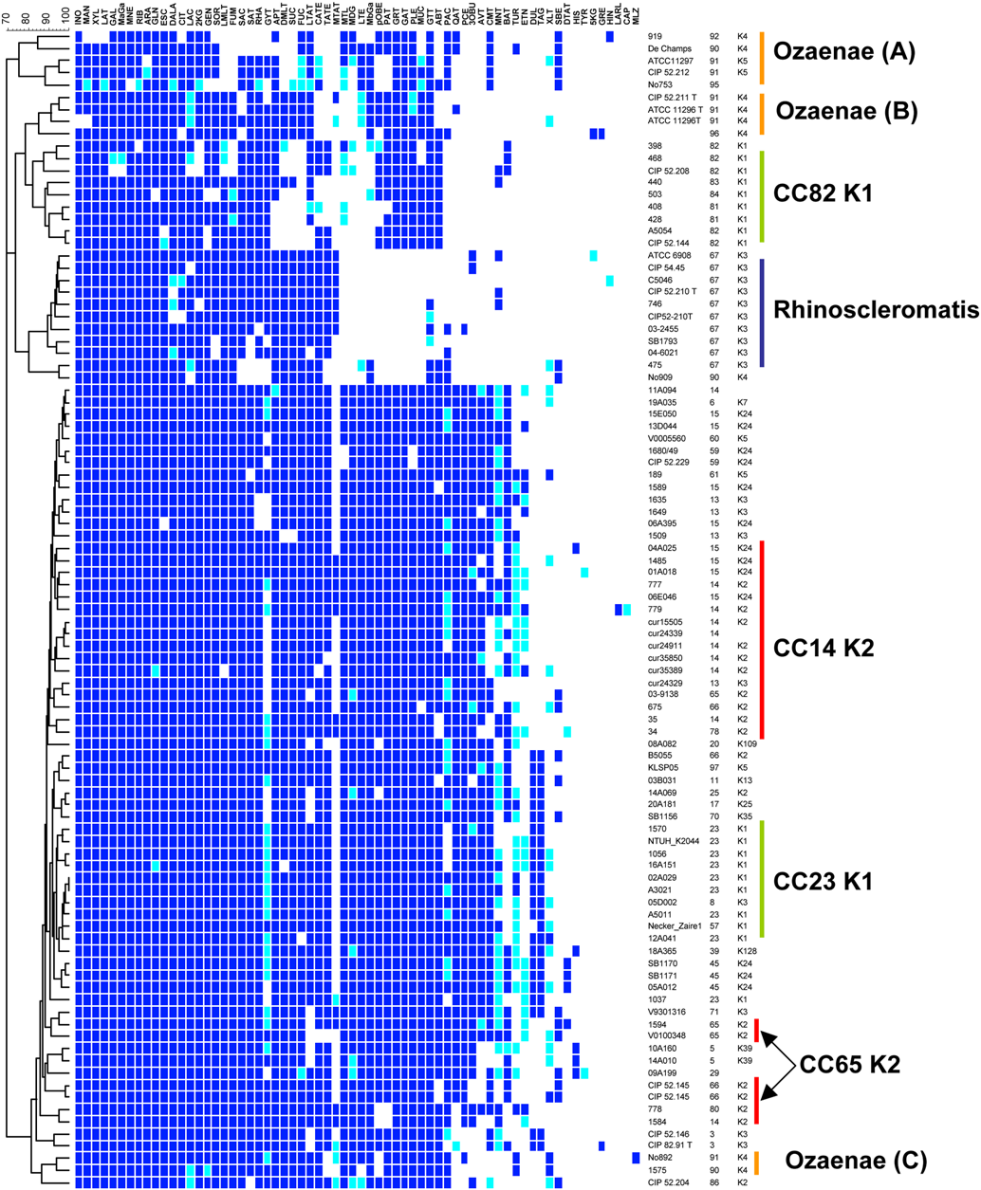
Biotype profiling of *K. pneumoniae* clones. Cluster analysis (simple matching coefficient) of *K. pneumoniae* isolates and reference strains based on metabolic profiles as assessed by biotype-100 strips. Codes above the column correspond to substrate code (Table S3). A blue square means the strain grew on the corresponding substrate as sole carbon source. Dark blue, growth was observed after two days; light blue, growth observed after four days. Note the strong homogeneity of biotype-100 profiles within clones. Three clones (Ozaenae, Rhinoscleromatis and CC82 K1) have lost the ability to utilize a number of substrates, including some common substrates between the three clones (see text). Note that three tests measure coloration, not growth: hydroxyquinoline-beta-glururonide (black color), tryptophane (brown color: hydrolysis into indole-pyruvic acid) and histidine (red color). The following substrates were utilized by all assayed strains: D-Glucose, D-fructose, D-trehalose, D-Melibiose, D-Raffinose, Maltotriose, Maltose, D-Cellobiose, 1-O-Methyl-B-D-glucoside, D-Arabitol, Glycerol, Adonitol, N-Acetyl-D-glucosamine, D-Gluconate, L-Alanine and L-Serine. The following substrates were always negative: hydroxyquinoline-beta-glucuronide, D-Lyxose, i-Erythritol, 3-O-Methyl-D-glucose, Tricarballylate, Tryptophan, Gentisate, 3-Hydroxybenzoate, 3-Phenylpropionate, Trigonelline, Betaine, Caprylate, Tryptamine, Itaconate, Propionate, 2-Ketoglutarate.

In order to determine whether virulent clones of *K. pneumoniae* are truly in the process of adapting to a pathogenic lifestyle, rather than simply representing classical *K. pneumoniae* strains with particular combinations of virulence factors, the ability to utilize 99 carbon sources was compared between representative isolates of the virulent clones and other *K. pneumoniae* isolates (**Figure 3**; **Table 3**). A total of 32 substrates were either utilized by all isolates (n = 16) or by none (n = 16, **Figure 3** **legend**); some of these substrates are useful for identification of the *K. pneumoniae* species [27]. However, the remaining substrates showed differences among *K. pneumoniae* strains. Interestingly, the pattern of carbon source utilization correlated closely with MLST-defined clones (**Figure 3**; **Table 3**). Clone Rhinoscleromatis showed a restricted substrate utilization pattern, with the distinctive loss of the ability to use seven substrates, including D-glucuronate and D-galacturonate (0% vs. 100%) and protocatechuate, an intermediate in the degradation of lignin. Isolates of CC82^K1^ also had a clearly distinctive pattern, in particular with the loss of L-fucose, D-(+)malate and succinate utilization. Clone Ozaenae isolates exhibited three groups of metabolic profiles (A, B and C on **Figure 3**), each of these consisting of the loss of a number of substrates, with trans-aconitate in common. Finally, the remaining isolates formed a large group that comprised K1 isolates of CC23^K1^, showing that these can be differentiated from CC82^K1^ by the utilization of several carbon sources (**Table 3**). In addition, CC23^K1^ were almost exclusive among *K. pneumoniae* isolates in using dulcitol and D-tagatose as sole carbon source, while they differed from the remainder of the large cluster by the loss of benzoate utilization. Differently, CC14^K2^ and CC65^K2^ both belonged to the large biotype cluster and were weakly distinguished, although L-sorbose utilization was found only in CC65.

**Table 3:** Utilization of carbon sources by *Klebsiella pneumoniae* clones (a).

	Substrate code		Clone					
		Rhinoscleromatis	Ozaenae	CC82K1	CC23K1	CC14K2	CC65K2	Other STs
No. strains:		9	12	9	10	21	8	26
Mean±SD of No. of positive substrates per strain:		47±2.4	50±5.9	48±2.2	66±1.4	65±1,8	65±1.8	65±3,4
*Carbon sources that discriminate among clones*:								
D-glucuronate	GRT	0	100	100	100	100	100	100
D-galacturonate	GAT	0	100	100	100	100	100	100
Palatinose	PLE	0	67	100	100	100	100	100
Protocatechuate	PAT	0	84	89	100	85	100	100
p-Hydroxybenzoate (4-Hydroxybenzoate)	pOBE	0	67	67	100	85	88	95
Mucate	MUC	0	67	78	100	100	88	100
trans-Aconitate	TATE	89	0	56	100	92	88	95
D(−) Ribose	RIB	100	100	0	100	100	100	100
α−L(−) Fucose	FUC	100	84	0	67	100	100	95
D(+) Malate	DMLT	100	50	11	67	100	100	95
Succinate	SUC	100	50	11	100	100	100	95
(−) Quinate	QAT	0	50	0	100	85	100	86
Maltitol	MTL	0	50	11	100	100	100	100
1-0-Methyl-α−D-glucopyranoside	MDG	0	50	0	100	100	75	81
m-Coumarate	CMT	0	50	0	100	39	100	86
Lactulose	LTE	0	33	0	100	100	100	95
L(+) Sorbose	SBE	11	50	0	0	0	88	29
1-0-Methyl-β-galactopyranoside	MbGa	11	84	0	100	100	100	100
DL-α-Amino-n-valerate( = 5-Aminovalerate)	AVT	0	0	0	100	62	50	62
DL-β-Hydroxybutyrate ( = 3-Hydroxybutyrate)	3OBU	22	0	0	100	100	63	81
Putrescine ( = Diaminobutane)	PCE	11	0	0	100	100	100	86
D-Tagatose	TAG	0	0	0	100	0	25	43

(a) % positive reactions at day 2.

On average, isolates of the largest cluster were able to utilize more carbon sources (65±3), whereas isolates of clone Rhinoscleromatis were those with the lowest metabolic abilities (47±2.4) (**Table 3**). CC82^K1^ and Ozaenae isolates (groups A, B and C together) used 48±2.2 and 50±5.9 substrates, respectively. It was striking that several substrates were lost in common by the three metabolically-restricted clones. For example, D-Malate and succinate were lost by Ozaenae (group A) and CC82^K1^, trans-aconitate was lost by Ozaenae groups A and B and by CC82^K1^, 1-O-Methyl-a-D-glucoside and lactulose were lost by CC82^K1^ and Rhinoscleromatis, whereas several substrates (e.g. 5-aminovalerate) were lost by the three groups. The loss of the same metabolic abilities indicates convergent evolution in these clones, possibly indicative of parallel specialization to a similar niche.

## Discussion

The population of *K. pneumoniae* appears to be characterized by a low level of nucleotide divergence among orthologous genes, contrasting with related species such as *S. enterica* and *E. coli*. This restricted polymorphism cannot be attributed to a biased sampling, as our dataset included isolates from the environment and animals, in addition to human isolates from different clinical sources and large geographic and temporal scales. The genetic distance that separates *K. pneumoniae* from its closest phylogenetic relatives (KpII and KpIV [40], [57]) calculated based on the same seven genes is nearly 4%. Therefore, the species *K. pneumoniae* may have undergone a bottleneck relatively recently, long after its separation from its closest relatives. Still, *K. pneumoniae* is much more diverse than notorious monomorphic pathogens such as *Y. pestis* or *S. enterica* serotype Typhi [58], [59].

A high number of distinct genotypes were disclosed by MLST despite restricted nucleotide polymorphism. Our analyses suggest that homologous recombination has more impact on sequence evolution than mutation, although it is difficult to obtain a reliable estimate of the recombination/mutation ratio with such a low level of polymorphism. A high recombination rate would shuffle polymorphisms among clones and lineages, generating many genotypes that can be distinguished by MLST. As a consequence, the clonal frame of *K. pneumoniae* clones will diversify more rapidly than it would by a purely mutational process, and the disclosed STs may not be highly stable over long periods of time.

Gene *gnd* was atypical by its high level of polymorphism. Because this gene is located between the *rfb* and *cps* operons responsible for the synthesis of the two major surface polysaccharides, the lipopolysaccharide and capsule, its evolution is probably highly influenced by the likely positive selection operating at these two neighboring loci, as demonstrated for *E. coli* or *Salmonella* [60], [61]. In addition, exchange of the *cps* operon between *E. coli* and *K. pneumoniae* was reported [60], and the divergent *gnd* alleles encountered in the present study clearly indicate incorporation into *K. pneumoniae* isolates of nucleotide sequences from other *Enterobacteriaceae* species.

Determining the phylogenetic relationships within a recombining species is difficult and may even be meaningless if recombination has erased the pattern of descent among strains. In particular, analysis based on allelic profiles can be misleading and may result in the clustering of unrelated STs into long straggly chains of genotypes [62]. One can therefore be suspicious about the true clonal link between isolates of CC17, which consists of chains of STs with distinct K-types, with the exception of some possibly meaningful terminal groupings such as three K5 STs (**Figure 1**).

Identification of clones within species with high rates of recombination is possible if these clones spread in the population [63], [64]. The fact that several clonal complexes disclosed herein are relatively homogeneous with respect to several features including K type, virulence factor content and metabolic profile, demonstrates that they correspond to clones, i.e. descend from a common ancestral strain from which they inherited their common properties. So far, K serotyping has been the dominant common language for recognition of related *Klebsiella* strains in epidemiological and virulence studies, but it was unknown whether isolates with the same K type belonged to single clones. Our data clearly reject this simple view. Indeed, most K-types (with the exception of K4) that were represented by several isolates were dispatched in unrelated STs. We could show that the shared K type resulted from horizontal transfer of the *cps* operon among these unrelated genotypes, generally with the co-transfer of the adjacent *gnd* gene. Therefore, knowledge of the K type provides unreliable prediction of clone identity. Given their close physical linkage, recombination between *gnd* and *cps* is probably unfrequent, and *gnd* sequencing could therefore be used as a proxy for K typing, which is technically demanding [50], [65], [66]. However, the finding of unrelated *gnd* sequences in the two major CCs of K2 isolates shows that this method would not be totally reliable.

Isolates with serotypes K1 to K4 were preferentially included in this study; therefore, our isolate collection does not reflect K type frequency in natural populations. Our selected collection allowed the discovery of six clones comprising isolates that are considered as particularly pathogenic based on clinical features in animal and humans and on experimental evidence [1], [32]–[35]. Our data provide the first evidence that the agent of rhinoscleroma on the one hand, and isolates recovered from cases of ozaena on the other hand, each correspond to a single clone. It is remarkable that these highly homogeneous clones include isolates that were isolated over a time span of several decades from several countries in Asia, Africa and Europe. Hence, these two pathogens, both involved in chronic infections, can be viewed as monomorphic pathogens, similar in this respect to e.g. *Mycobacterium leprae* [67]. Nevertheless, isolates of clone Ozaenae appear to be slightly more heterogeneous than Rhinoscleromatis based on MLST data, K type and biotype. Ozaenae isolates have also been implicated in distinct types of infections such as bacteremia, urinary tract infections [68] or splenic abscess [69], and were variable for the presence of several virulence factors. These observations may reflect a more diverse lifestyle for Ozaenae than for the intracellular human-restricted pathogen Rhinoscleromatis.

“*K. ozaenae*” and “*K. rhinoscleromatis*” could not be separated from *K. pneumoniae* by DNA relatedness [70]. For this reason, *K. ozaenae* and *K. rhinoscleromatis* were treated as subspecies of *K. pneumoniae* in the early editions of the Bergey's Manual [18], [71]. However, these two clones appear to have evolved from the genetic pool taxonomically regarded as *K. pneumoniae* subsp. *pneumoniae*, which does not form a phylogenetic lineage distinct from the other two subspecies (this study and [40]). Therefore, it is appropriate to consider isolates associated to rhinoscleroma and ozaena as clones of *K. pneumoniae* that acquired particular pathogenic properties, rather than separate phylogenetic entities that deserve subspecies status. Our data do not indicate a close affiliation of clone Rhinoscleromatis with clone Ozaenae. The uncultivable agent of donovanosis, or granuloma inguinale, has been included in the genus *Klebsiella* as *K. granulomatis* [26], [72]. Its *phoE* sequence [26], allele *phoE*-1, was encountered in several *K. p. subsp. pneumoniae* STs (including CC14^K2^), and is distinct from *phoE*-15 found in Rhinoscleromatis. Despite the similarities in the pathologies they cause [21], [25], [26], it was thus not possible to equate *K. granulomatis* with clone Rhinoscleromatis, but a close evolutionary link cannot be excluded. In any case, *phoE* data indicate that *K. granulomatis* does not represent a distinct genomic species, but instead belongs to *K. pneumoniae* as well.

This study demonstrates for the first time that K1 isolates that cause PLA are genetically distinct from K1 isolates from cases of respiratory infections and septicemia. Even though our identification of CC23^K1^ as the only clone associated with PLA is based on only four PLA-causing isolates, this result is fully consistent with a previous report based on a worldwide collection [38]. Recent progress stimulated by the emergence of *K. pneumoniae* PLA has provided important clues as to the bacterial factors involved in this infection [30], [31], [52], [73]. Our PCR tests show that among the genetic factors that have been associated with K1 PLA strains, only *allS* appears to be totally specific for this pathogen. In addition, we show for the first time that *allS* is not universally present in K1 strains [30], [74]. Our data provide the novel observation that CF29K is particularly prevalent in this clone. CF29K corresponds to adhesin CS31A found in *E. coli* strains and involved in human diarrhea and in septicemia in calves [55]. Our data suggest that this factor could either be directly implicated in the pathogenesis of PLA, or linked to another pathogenicity factor on the 185 kb plasmid that harbors gene *cf29A* [75]. In agreement with others [76], we found that *magA* is present in K1 isolates not involved in PLA and should thus not be considered as a marker of PLA-causing isolates [52]. The respiratory or blood origin of most CC82^K1^ isolates, together with previous reports of the frequent implication of K1 strains in Friedländer's pneumonia, is consistent with this clone being a prominent agent of this severe form of pneumonia. The existence of two K1 groups that differ by their pathological potential is of high relevance for understanding the bacterial determinants of PLA and acute pneumonia caused by *K. pneumoniae*. K2 isolates also clearly appear to be distributed into several unrelated genotypes. For both K1 and K2 serotypes, we could show that the pathogenic potential of strains depends on their genotype, rather than on their K type. CC14^K2^ comprises isolates for serotypes K2 and K24, but we did not observe any difference in virulence gene content or in virulence to mice between CC14^K2^ members of both serotypes. In contrast, some virulence factors distinguished CC14^K2^ (including its K24 isolates) and CC65^K2^, e.g. gene *kfu* (100% vs. 0%, respectively). These results show that at least for K1 and K2 isolates, the clonal complex is a better predictor of virulence gene content and of virulence to mice than the K type, and that previous associations of virulence factors with K-types [74], [77] should be revisited by analysis of isolates from distinct CCs. Thus, even if the capsular polysaccharide is a prominent pathogenicity determinant, the long-held belief that K type is predictive of virulence should be discontinued.

The nature of *K. pneumoniae* pathogenic clones and their history of interaction with their animal and human hosts, including possible specialization, remain largely unknown. Our biotype data demonstrated that the three clones Rhinoscleromatis, Ozaenae and CC82^K1^ have each lost several metabolic abilities, some of which in common, probably by parallel evolution. It has long been recognized that the two former clones are biotypes of *K. pneumoniae* with less nutritional versatility [18], [27] and together with some K1 strains, require specific factors for growth [27]. To our knowledge, Rhinoscleromatis, Ozaenae and K1 isolates have neither been reported from the environment, nor in intestinal carriage, and it is perhaps significant that several substrates that are not utilized by these clones belong to plant product degradation pathways. We hypothesize that these three clones are engaged in evolutionary specialization to a restricted ecological niche, possibly represented by the upper respiratory tract of humans. A restriction in ecological niche may in turn reduce the opportunity for encounter with other *K. pneumoniae* strains. The intracellular lifestyle of Rhinoscleromatis provides the most achieved example, and this pathogen may now be evolving independently from its ancestral species *K. pneumoniae*. Consistent with this hypothesis, Rhinoscleromatis and Ozaenae were genetically the most distinct of the 117 STs (**Figure 1**), which had in general no more than three allelic mismatches among themselves. This observation suggests that these two clones are less frequently involved in allelic exchange with other strains. Finally, it is interesting to notice that the gene coding for the adhesin MrkD was undetected specifically in CC82^K1^ and Ozaenae, also suggestive of niche reduction. In contrast, the typical biotype profile of the PLA-associated CC23^K1^ does not suggest ecological specialization. Hence, the acquisition by this clone of its particular set of virulence determinants is possibly recent in time, consistent with epidemiological data [12], and the pathogenicity of clone CC23^K1^ may be uncoupled from any particular adaptation to humans. Infection of the liver is believed to take place from the intestine. Because liver infection and metastasis to the eye and brain are unlikely to provide any specific selective advantage to this clone, pathogenesis can be viewed as accidental. Given that the natural habitat of this clone is probably indistinct from its non-virulent ancestor, keeping an intact metabolic versatility may be a key requirement for successful competition of this clone with other generalist *K. pneumoniae* clones. It is therefore unlikely that reductive evolution by specialization will be observed in this important emerging clone.

## Materials and Methods

### Bacterial isolates

A total of 235 *K. pneumoniae* reference strains or isolates were included in this study (**Table S1**). Capsular (K) serotypes K1 to K4 were included preferentially in order to estimate their genetic diversity. The collection included 25 isolates with serotype K1 from cases of pyogenic liver abscess (n = 4), other clinical sources (n = 17) and reference strains (n = 4). Nineteen K2 isolates, 16 *K. pneumoniae* subsp. *rhinoscleromatis* isolates (all being K3) and 14 *K. pneumoniae* subsp. *ozaenae* (12 K4 and 2 K5) were included. For comparison purposes, we included K3 (n = 10) and K5 isolates (n = 4) of *K. pneumoniae* subsp. *pneumoniae*. Type strains of the three subspecies and reference strains of serotypes K1 to K5 as well as laboratory strain KP52.145, were included. In some cases (**Table S1**, column ‘probable duplicate’), two or more subcultures of the same original strain were included, because they were obtained from different sources (e.g., the Orskov collection of K-type reference strains, the Collection de l'Institut Pasteur [CIP] and the ATCC). This is due to the fact that the K-type reference strain and the taxonomic type strain or other laboratory strains are sometimes derived from the same initial strain.

The remaining isolates were included to represent different sources, without consideration of their K type. Most isolates were of human clinical origin. For comparison purposes, we collected 13 isolates from the environment and 18 from fecal samples using a selective medium based on citrate and inositol [78], and gathered 30 horse isolates and 8 other animal isolates from previous studies [49], [79]–[81]. The 67 isolates from nosocomial infections previously analyzed [82] were included. Isolates originated from 20 countries from Europe, North America, Asia and Africa. The most represented countries were France and the Netherlands (**Table S1**).

### Species and subspecies identification and biotyping

Isolates were initially identified as *Klebsiella pneumoniae sensu stricto*, *K. pneumoniae* subsp. *rhinoscleromatis* or *K. pneumoniae* subsp. *ozaenae* using standard, recommended biochemical tests [27]. Identification of the later two subspecies was controlled by capsular serotyping using the capsular swelling method and using the following biochemical tests: Voges-Proskauer, urease, ONPG, lysine decarboxylase, citrate, malonate, and gas production. Isolates could be identified as belonging to *K. pneumoniae sensu stricto*, i.e., phylogenetic group KpI [40], based on phylogenetic clustering of the seven MLST genes and *gyrA* [40], using KpII-A, KpII-B [57], KpIII [40] and *K. oxytoca*, *K. planticola* and *K. terrigena* isolates for comparison.

Re-identification and biotyping of isolates at the species level (or subspecies level within *K. pneumoniae*) was performed using Biotype-100 strips (BioMérieux, Marcy l'Etoile, France), which contain 99 substrates in cupules [27]. Minimal medium 1 was used and isolates were identified using software Recognizer (P.A.D. Grimont, Institut Pasteur) against the *Enterobacteriaceae* database constructed in the laboratory (version 2000). Substrates that were particularly useful for species discrimination were m-coumarate, gentisate, histamin, 3-hydroxybenzoate, D-melezitose, 3-O-methyl-D-glucose, and tricarballylate [27]. Minimal medium 2 was used for isolates of *K. pneumoniae* subsp. *rhinoscleromatis* or *K. pneumoniae* subsp. *ozaenae* [27]. Reproducibility of biotype-100 profiles was controlled by inclusion of strain ATCC 13883^T^ in each batch and by the independent analysis of synonymous strains (**Table S1**; **Figure 3**).

### Capsular serotyping

Serotyping was determined by the capsular swelling method [49], [79], [80], and the K-type of some isolates were controlled by the agglutination method [65]. The K-serotype of the type strains and reference strains was known prior to this study.

### *cps* PCR-RFLP (molecular serotyping)

The determination of the C-pattern was determined as previously described [50]. A reference C-pattern database was constituted by the C-patterns obtained for the 77 reference strains of the International serotyping scheme and for the study isolates for which the K-type was determined by classical serotyping. C-patterns that were encountered in isolates of defined capsular type were labeled with ‘C’ followed by the number capsular (e.g., C2) followed by a letter denoting the successive banding patterns found for isolates of this serotype (e.g., C2a, C2b …). Some isolates were analyzed by *cps* PCR-RFLP but not by classical serotyping. For the C-patterns that had a match in the reference database, the same K-type as that of the reference strain(s) was inferred. For the remaining isolates, the obtained C-pattern had no match in the reference database; these C-patterns were numbered consecutively, starting at C100 (**Table S1**).

### Multilocus Sequence Typing (MLST)

MLST was performed as previously described [82] with the following modification: universal sequences were added upstream of each forward (GTT TTC CCA GTC ACG ACG TTG TA) and reverse (TTG TGA GCG GAT AAC AAT TTC) primers. All amplifications were performed at 50°C, and sequences were obtained using the two universal sequencing primers given above. Further details are available on the *K. pneumoniae* MLST web site (www.pasteur.fr/mlst).

### *gnd* gene sequencing

The sequence of a 360-bp portion of the *gnd* gene was established on both strands by using primers gnd-1F (TGA AGC AGC AAA CAA AGG TAC) and gnd-8R (TCA TCG GCG ATC TGC TTA AAG T), which amplify an internal portion of 457 bp of the gene. The annealing temperature was 46°C (30 cycles of 30 sec, 94°C; 30 sec., 46°C; 30 sec., 72°C, followed by 1 min at 72°C). When amplification failed, primer gnd-2 (ACA TCA CGC AGC GCC TGC TGA T) was used instead of gnd-8R, with 50°C as annealing temperature. Sequencing primers were gnd-9, TGA TGA (A/G)GC nGC (A/c)AA CAA AGG TAC, and gnd-10, TCA TCa GC(a/G) ATC TG(C/t) TTG AAG Ta(c/t).

### Virulence PCR

PCR assays were performed to check for the presence of 10 genes that have previously been associated with virulence in *K. pneumoniae*. Target genes, primers used and specific annealing temperature of PCR are given in **Table S2**. After 5 min at 94°C, there were 35 cycles of 94°C, 30 sec.; annealing temperature, 30 sec.; and 72°C, 1 min. followed by a final elongation of 1 min at 72°C. Strains NTUH-K2044, KP52145 and MGH 78578 [31], [34], [52], [83] were used as PCR controls. PCR products from several STs were systematically sequenced to control that the amplified PCR products corresponded to the expected gene.

### Infection of mice

Female Balb/cJ mice were purchased from R. Janvier Breeding Center (Le Genest St. Isle, France) and housed under standard conditions of feeding, light and temperature with free access to food and water. Experiments were performed according to the Institut Pasteur guidelines for laboratory animals husbandry. Seven to eight weeks-old mice were first anesthetized, with 80 microliters intramuscular injection of ketamine (Imalgene, 31.25 mg/kg, Merial) and Acepromazine (Calmivet, 1.5 mg/kg, Vetoquinol) and then infected by inoculation of 20 microliters of bacteria suspension (10^6^ bacteria) into their right nostril. Eight mice per strain were infected. The number of surviving mice was monitored every day during twelve days.

### Data analysis

For each MLST locus, an allele number was given to each distinct sequence variant (confirmed by at least two chromatogram traces), and a distinct sequence type (ST) number was attributed to each distinct combination of alleles at the seven genes. Allele and profile numbers were incremented successively in the order in which they were discovered. In order to define the relationships among isolates at the microevolutionary level, we performed allelic profile – based comparisons using a minimum spanning tree (MStree) analysis with the BioNumerics v5.10 software (Applied-Maths, Sint Maartens-Latem, Belgium). MStree analysis links profiles so that the sum of the distances (number of distinct alleles between two STs) is minimized [84]. Isolates were grouped into clonal complexes (clonal families), defined as groups of profiles differing by no more than one gene from at least one other profile of the group [85]. Accordingly, singletons were defined as STs having at least two allelic mismatches with all other STs.

Split decomposition analysis was performed using SplitsTree version 4.10 [86], [87]. Neighbor-joining tree analysis was performed using MEGA v4 [88]. Nucleotide diversity indices were calculated using DNAsp v4 [89]. ClonalFrame analysis [46] was performed with 50,000 burn-in iterations and 100,000 subsequent iterations.

The relative contribution of recombination and mutation on the short term was calculated using eBURST and the clonal diversification method [90], [91]. For each pair of allelic profiles that differ by a single allelic mismatch (single locus variants, or SLVs), the number of nucleotide changes between the alleles that differ is counted. A single nucleotide difference is considered to be likely caused by mutation, whereas more than one mutation in the same gene portion is considered to derive from recombination, as it is considered unlikely that two mutations would occur on the same gene while the other genes remain identical. No correction was made for single nucleotide differences possibly introduced by recombination.

The population recombination rate was estimated by a composite-likelihood method with LDhat [92]. LDhat employs a parametric approach, based on the neutral coalescent, to estimate the scaled parameter 2*N*_e_ *r* where *N_e_* is the effective population size, and *r* is the rate at which recombination events separate adjacent nucleotides. The crossing-over model L was used for the analysis of biallelic sites, with frequency of the less frequent allele >0.1.

### Nucleotide sequences

Sequences generated in this study are available at www.pasteur.fr/mlst for the seven MLST genes. In addition, *gnd* alleles have been deposited in GenBank/EMBL/DDBJ databases under the accession numbers FJ769917-FJ769969.

## Supporting Information

Table S1 Strains. Characteristics of the 235 strains included in the study.
(0.13 MB XLS)

Table S2 Primers used for virulence genes PCR.
(0.04 MB XLS)

Table S3 Carbon sources assayed with the Biotype-100 strips.
(0.01 MB PDF)
