# Supplementary material for: Virulent Clones of Klebsiella pneumoniae: Identification and Evolutionary Scenario Based on Genomic and Phenotypic Characterization
Source: PLoS One. 2009 Mar 25;4(3):e4982. doi: 10.1371/journal.pone.0004982 (PMC2656620; doi:10.1371/journal.pone.0004982)
Supplement: Table S3 — Carbon sources assayed with the Biotype-100 strips. (0.01 MB PDF) [file pone.0004982.s003.pdf]

Table S3. Carbon sources assayed using the Biotype-100 strips.

Position	Substrate (# position)	Substrate code
1	D-Glucose (# 1)	GLU
2	D-Fructose (# 2)	FRU
3	D-Galactose (# 3)	GAL
4	D-Trehalose (# 4)	TRE
5	D-Mannose (# 5)	MNE
6	L-Sorbose (# 6)	SBE
7	D-Melibiose (# 7)	MEL
8	Sucrose (# 8)	SAC
9	D-Raffinose (# 9)	RAF
10	Maltotriose (# 10)	MTE
11	Maltose (# 11)	MAL
12	Lactose (# 12)	LAC
13	Lactulose (# 13)	LTE
14	1-O-Methyl-B-galactoside (# 14)	MbGa
15	1-O-Methyl-a-galactoside (# 15)	MaGa
16	D-Cellobiose (# 16)	CEL
17	Gentiobiose (# 17)	GEN
18	1-O-Methyl-B-D-glucoside (# 18)	MbGu
19	Esculin (black color) (# 19)	ESC
20	D-Ribose (# 20)	RIB
21	L-Arabinose (# 21)	ARA
22	D-Xylose (# 22)	XYL
23	Palatinose (# 23)	PLE
24	L-Rhamnose (# 24)	RHA
25	L-Fucose (# 25)	FUC
26	D-Melezitose (# 26)	MLZ
27	D-Arabitol (# 27)	DARL
28	L-Arabitol (# 28)	LARL
29	Xylitol (# 29)	XLT
30	Dulcitol (# 30)	DUL
31	D-Tagatose (# 31)	TAG
32	Glycerol (# 32)	GLY
33	myo-Inositol (# 33)	INO
34	D-Mannitol (# 34)	MAN
35	Maltitol (# 35)	MTL
36	D-Turanose (# 36)	TUR
37	D-Sorbitol (# 37)	SOR
38	Adonitol (# 38)	ADO
39	HQ-B-glucuronide (# 39)	HBG
40	D-Lyxose (# 40)	LYX
41	i-Erythritol (# 41)	ERY
42	1-O-Methyl-a-D-glucoside (# 42)	MDG
43	3-O-Methyl-D-glucose (# 43)	3MDG
44	D-Saccharate (# 44)	SAT
45	Mucate (# 45)	MUC
46	L-Tartrate (# 46)	LTAT
47	D-Tartrate (# 47)	DTAT
48	meso-Tartrate (# 48)	MTAT
49	D-Malate (# 49)	DMLT

50	L-Malate (# 50)	LMLT
51	cis-Aconitate (# 51)	CATE
52	trans-Aconitate (# 52)	TATE
53	Tricarballylate (# 53)	TTE
54	Citrate (# 54)	CIT
55	D-Glucuronate (# 55)	GRT
56	D-Galacturonate (# 56)	GAT
57	2-Ketogluconate (# 57)	2KG
58	5-Ketogluconate (# 58)	5KG
59	Tryptophan (orange color) (# 59)	TRY
60	N-Acetyl-D-glucosamine (# 60)	NAG
61	D-Gluconate (# 61)	GNT
62	Phenylacetate (# 62)	PAC
63	Protocatechuate (# 63)	PAT
64	4-Hydroxybenzoate (# 64)	pOBE
65	Quinate (# 65)	QAT
66	Gentisate (# 66)	GTE
67	3-Hydroxybenzoate (# 67)	mOBE
68	Benzoate (# 68)	BAT
69	3-Phenylpropionate (# 69)	PPAT
70	m-Coumarate (# 70)	CMT
71	Trigonelline (# 71)	TGE
72	Betaine (# 72)	BET
73	Putrescine (# 73)	PCE
74	4-Aminobutyrate (# 74)	ABT
75	Histamine (# 75)	HIN
76	DL-Lactate (# 76)	LAT
77	Caprate (# 77)	CAP
78	Caprylate (# 78)	CYT
79	L-Histidine (pink color) (# 79)	HIS
80	Succinate (# 80)	SUC
81	Fumarate (# 81)	FUM
82	Glutarate (# 82)	GRE
83	DL-Glycerate (# 83)	GYT
84	5-Aminovalerate (# 84)	AVT
85	Ethanolamine (# 85)	ETN
86	Tryptamine (# 86)	TTN
87	D-Glucosamine (# 87)	GLN
88	Itaconate (# 88)	ITA
89	3-Hydroxybutyrate (# 89)	3OBU
90	L-Aspartate (# 90)	APT
91	L-Glutamate (# 91)	GTT
92	L-Proline (# 92)	PRO
93	D-Alanine (# 93)	DALA
94	L-Alanine (# 94)	LALA
95	L-Serine (# 95)	SER
96	Malonate (# 96)	MNT
97	Propionate (# 97)	PROP
98	L-Tyrosine (# 98)	TYR
99	2-Ketoglutarate (# 99)	2KT
